# Circulating microparticles and activated platelets as novel prognostic biomarkers in COVID-19; relation to cancer

**DOI:** 10.1371/journal.pone.0246806

**Published:** 2021-02-22

**Authors:** Asmaa M. Zahran, Omnia El-Badawy, Wageeh A. Ali, Zainab Gaber Mahran, Essam Eldeen M. O. Mahran, Amal Rayan

**Affiliations:** 1 Department of Clinical Pathology, South Egypt Cancer Institute, Assiut University, Assiut, Egypt; 2 Department of Medical Microbiology and Immunology, Faculty of Medicine, Assiut University, Assiut, Egypt; 3 Diagnostic and Interventional Radiology Department, Faculty of Medicine, Assiut University, Assiut, Egypt; 4 Department of Tropical Medicine and Gastroenterology, Faculty of Medicine, Assiut University, Assiut, Egypt; 5 Clinical Oncology Department, Faculty of Medicine, Assiut University, Assiut, Egypt; Saskatoon Cancer Centre and College of Medicine, University of Saskatchewan, CANADA

## Abstract

**Background and aim:**

The study aimed to determine whether the MPs levels and platelet activation are affected by the COVID-19 infection in both malignant and non-malignant patients compared to healthy individuals and define their contribution to the COVID-19 associated coagulopathy and the relation of these MPs to other hematologic parameters.

**Patients and methods:**

We recruited 23 malignant patients with reverse transcription polymerase chain reaction (RT-PCR) positive COVID-19, also, 19 COVID-19 non-malignant patients, and 20 healthy volunteers were also enrolled for comparison. Blood samples were collected from patients and healthy donors into 5 mL vacutainer tube containing 3.5% buffered sodium citrate solution for measurement of total microparticles (TMPs), platelet microparticles (PMPs), endothelial microparticles (EMPs), CD62 activated platelets, and CD41 platelet marker.

**Results:**

COVID-19 malignant patients had significantly lower hemoglobin and platelets compared to COVID non-malignant ones, while they had significantly higher C-reactive protein, LDH, AST, Albunim, creatinine, and prognostic index (PI) compared to COVID-19 non-malignant patients. significant accumulations of TMPs, PMPs, EMPs, and activated platelets in COVID-19 affected patients compared to healthy controls. TMPs, and EMPs were significantly accumulated in COVID-19 malignant compared to COVID-19 non-malignant patients with no significant difference in PMPs between both.

**Conclusion:**

Circulating MPs and activated platelets may be promising novel prognostic biomarkers capable of identifying potentially severe COVID-19 patients who require immediate care especially in cancer patients.

## Introduction

Microparticles are a diverse group of bioactive small-sized vesicles (100–1000nm) that can be found in body fluids and blood after activation, necrosis, or apoptosis of almost all cells [1]. It is hypothesized that they participate in intercellular communication and play a major role in homeostasis under physiological conditions. In addition to their physiological roles, there has become uprising evidence of their association with multiple diseases. For that, microparticles (MP) detection has become of great interest in the last decade as a result of cumulative evidence of not only their significant role in multiple cellular processes but also other diseases.

Although most MPs in human blood originate from platelets, they also arise from erythrocytes, leukocytes, endothelial cells, smooth muscle cells, and cancer cells. Subsequently, MPs composition displays their cellular membranous origin including various cytoplasmic components, adhesion molecules, and antigens that distinguish the cell of origin and their stimuli [2]. The exact mechanisms of the formation of MPs are still unclear. Any cell can release MP such as tumor cells, smooth muscle, synovial cells, in addition to circulating blood cells, those of lymphoid origin express CD4, CD8, or CD3, while those of platelet origin CD41, and CD42a, and those of endothelial origin express on their surfaces CD144 or CD146 [3, 4].

Venous thrombo-embolism complicates many cancers such as pancreatic and breast cancers, and different chemotherapy and hormonal treatments, and it may be associated with cancer progression [5], MPs have been reported in almost all thromboembolic diseases because they contain procoagulant phospholipids [6].

Interactions between platelets and endothelium can be facilitated by numerous adhesions molecules and ligands. P-selectin (CD62 marker) expressed by platelets has a crucial role in linking between homeostasis and inflammation [7, 8].

Furthermore, platelet-derived MPs (PMPs) promote angiogenesis, invasion, and metastasis in lung [9] and breast cancers [10]. On the other hand, endothelial-derived MPs (EMPs) were correlated with the clinical outcomes of patients with hepatocellular carcinoma treated with liver transplantation [11], end-stage non small-cell lung cancer [12], and glioblastoma multiforme [13].

Recently, it has come to focus that the new corona virus disease 2019 (COVID 19) caused by the novel Corona virus SARS-CoV-2 has been associated with abnormalities in coagulation markers. It is concluded that one of the most significant prognostic factors, in patients with COVID-19, is coagulopathy and is accompanied by increased mortality and admission to critical care units [14].

The most commonly observed coagulopathy associated with COVID-19 is characterized by initially increased D-dimer and fibrinogen levels while prolonged prothrombin time, and partial thromboplastin time with decreased platelet count were uncommon at that initial presentation. Hence, D-dimer and fibrinogen levels were used as a screening test to decide which patients required critical care [15].

The study aimed to determine whether the MPs levels and platelet activation are affected by the COVID-19 infection in both malignant and non-malignant patients compared to healthy individuals, and define their contribution to the COVID-19 associated-coagulopathy, and relation of these MPs to other hematologic parameters.

## Patients and methods

This study was carried out at South Egypt Cancer Institute, and Assiut University Hospital, and was approved by institutional review board of ethical committee of faculty of medicine, Assiut University (IRB no:17300436).Written informed consent was taken from all subjects after explaining the study objectives to them in a safe manner. All intervention procedures were done by sterilized and safe maneuvers, and all research tests were conducted by scientifically qualified and trained personnel.

### Study population

We recruited 23 malignant patients with reverse transcription polymerase chain reaction (RT-PCR) positive COVID-19 isolated at Clinical Oncology Department, also, 19 non malignant patients isolated at Al Rajhi hospital of AssiutUniversity for the same issue during a period of two months (June and July/2020), and 20 healthy volunteers were also enrolled for comparison. We couldn’t recruit patients subjected to the protocol of home isolation and treatment which limited the number of patients enrolled.

We included malignant patients completed their chemotherapy and radiotherapy treatment courses, patients under targeted treatments (three patients were on this type of treatment; one with hepatocellular carcinoma and under sorafenib treatment, one with metastatic breast cancer under pulbociclib/Fulvestrant/zoladex, and another one with renal cell carcinoma under sunitinib), patients with hematologic malignancies who completed their protocols of treatment, and female patients who were under hormonal therapy, malignant patients recruited in this study included one with HCC, one with RCC, one with osteosarcoma, 3 patients with esophageal cancer, 2 patients with nasopharynx, one patient with rectal cancer, one with HD, one with bladder cancer, 2 patients with pancreatic adenocarcinoma, 3 patients with NHL, and 7 patients with breast cancer.

We excluded patients with uncontrolled active malignancy who were in need for continuation of treatment protocols wether chemotherapy or radiotherapy, patients admitted at ICU for complications of their malignancies or treatments, and patients with other concomitant infections.

### Clinical methodology

These patients were clinically evaluated for their manifestations of covid; including the degrees and duration of fever, cough and its type, the presence or absence of diarrhea, anosmia, grades of dyspnea, contact relation to COVID-19 cases, and their radiological manifestations.

Grades of dyspnea were applied according to CTC-AE version 4.0 [16]; grade 0; No dyspnea, grade 1; Shortness of breath with moderate exertion, grade 2; Shortness of breath with minimal exertion; limiting instrumental activities of daily living (ADL), grade 3; Shortness of breath at rest; limiting self care ADL, grade 4; Life-threatening consequences; urgent intervention indicated, grade 5; death.

The chest CT findings commonly reported for COVID-19 were ground glass opacity (GGO), crazy-paving pattern, and pulmonary consolidation that were based on the standard glossary for thoracic imaging reported by the Fleischner Society [17, 18]. In all cases, a semi-quantitative CT severity scoring proposed by Pan et al [19] was calculated per each of the 5 lobes of both lungs considering the extent of anatomic involvement as follows: 0; no involvement; 1;< 5% involvement, 2; 5–25% involvement, 3; 26–50% involvement, 4; 51–75% involvement, and 5;>75% involvement (S1, S2, S3 and S4 Figs). the resulting global CT score was the sum of each individual lobar score and (0 to 25). When present, related features such as fibrosis, subpleural lines, reversed “halo sign,” pleural effusion, and lymphadenopathy were also described. Because of small number of cases recruited in this study, a simpler scoring was applied based mainly on GGO where no involvement is considered score 0, mild affection <25% of both lung fields (corresponding to scores 1, 2), moderate affection >25%-<75% of both lung fields (corresponding to scores 3, 4), and severe affection >75% of both lung fields (corresponding to score 5).

Patients were evaluated for routine complete blood pictures, blood chemistries, C-reactive protein (CRP), serum ferritin, lactate dehydrogenase (LDH), D-dimer, glycated hemoglobin(HbA1C) was evaluated; where those with values<5.5 were considered non diabetic, those with values 5.5–6.5 were considered controlled diabetic, and those with values >6.5 were considered uncontrolled diabetic.

Several inflammatory indices were calculated including, Neutrophil to lymphocyte ratio (NLR); calculated by dividing absolute neutrophilic count by absolute lymphocytic count, Platelet to lymphocyte ratio (PLR); calculated by dividing absolute platelet count by absolute lymphocytic count, Platelet to neutrophil ratio (PNR); calculated by dividing absolute platelet count by absolute neutrophil count, Prognostic index (PI); was determined as follows: PI 2; both CRP >1.0 mg/dl and white cell count >11 ×10^9^/l, PI 1;either CRP >1.0 mg/dl or white cell count >11 ×10^9^/l, but not both, and PI 0; no abnormality [20].

Blood and fluid cultures before flow cytometry, were done to exclude other concomitant viral and bacterial infections.

Blood samples were collected from patients and healthy donors into 5 mL vacutainer tube containing 3.5% buffered sodium citrate solution for measurement of TMPs, PMPs, EMPs, CD62 activated platelets, and CD41 platelet marker.

#### Microparticles isolation and characterization

In order to isolate the MPs, cells were removed by centrifugation for 20 min at 1550 ×g at 20°C within 15 min after collection. Then 250 μL of plasma was centrifuged for 30 min at 18,800 ×g at 20°C. After centrifugation, the supernatant was removed and the pellet was resuspended in phosphate-buffered saline (PBS) and centrifuged for 30 min at 18,800 ×g at 20°C. The supernatant was removed again and MPs pellet was resuspended in PBS.

**Fig 1:**
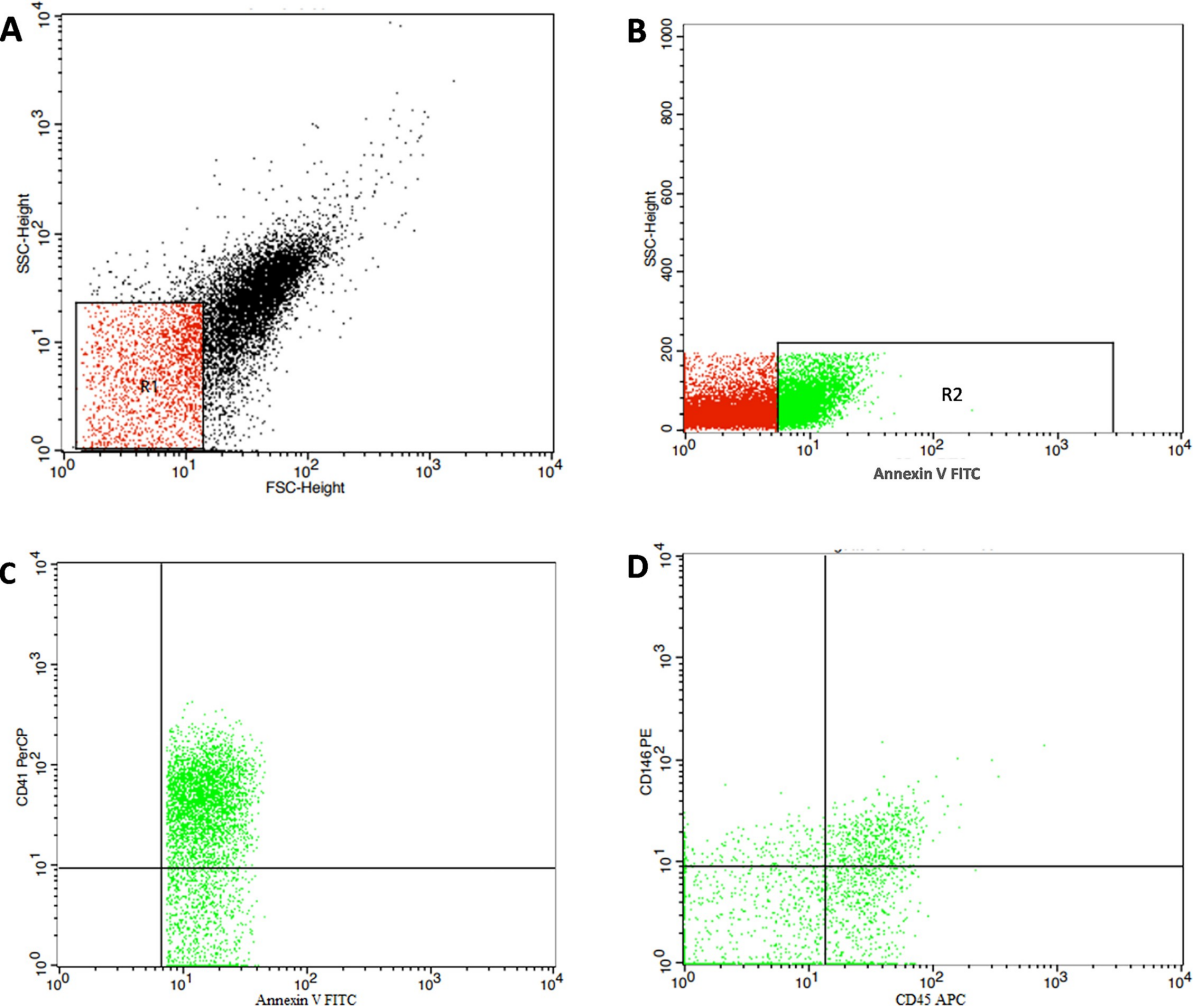
Flow cytometry gating strategy of microparticles (MPs). A: The MPs were identified in a forward/side scatter histogram (R1) using calibrated beads. B: The expression of annexin V was then assessed within the defined MP population, and the annexin V-positive MPs (total MPs) were quantified (R2). C & D: Total MPs were further analyzedfor the expression of CD41, CD45, and CD146 to detect the platelet MPs (CD41+ MPs) and the endothelial derived MPs (CD45-CD146+ MPs).

Flow cytometric analysis was used to quantify and characterize MPs. Five μL of MPs sampleswere diluted in 35 μLPBS containing 2.5 mM CaCl_2_. The samples were thenincubated for 20 min at room temperature in the dark with 5 μL of fluoroisothiocyanate (FITC)-conjugated annexin V, IQP-116F (IQ products, Netherland) and 5 μL of phycoerythrin (PE) conjugated CD146 Catalog No.550315 (Becton Dickinson Biosciences, USA) and peridinium-chlorophyll-protein (Per-CP) conjugated CD41 PC-309-T100 (EXBIO Praha, a.s, Vestec, Czech Republic), and allophycocyanin (APC)-conjugatedCD45 Catalog No.555485 (Becton Dickinson Biosciences, USA). After incubation, PBS/calcium buffer was added and the samples were analyzed on a Fluorescence Activated Cell Sorter (FACS) Calibur flow cytometer with Cell Quest software (Becton Dickinson Biosciences, USA). Fifty thousand events were analyzed, and MPs were reported as a percentage of the total events. Anti-human IgG was used as an isotype-matched negative control for each sample. Microparticles were identified on the basis of their forward scatter compared with 1.0 μm reference calibration beads of 1.0 μm to calibrate the microparticle size range (Latex beads, amine-modified polystyrene, fluorescent red aqueous suspension, 1.0 μm mean particle size, Sigma-Aldrich ChemieGmbHMunich, Germany) and positivity for annexin V. Microparticles express phosphatidyl serine, which is detected by annexin V labeling. Microparticles subpopulations were identified by their ability to bind cell-specific monoclonal antibodies. Endothelial MPs are CD146+ CD45- and platelet MPs are CD41+, "Fig 1".

**Fig 2:**
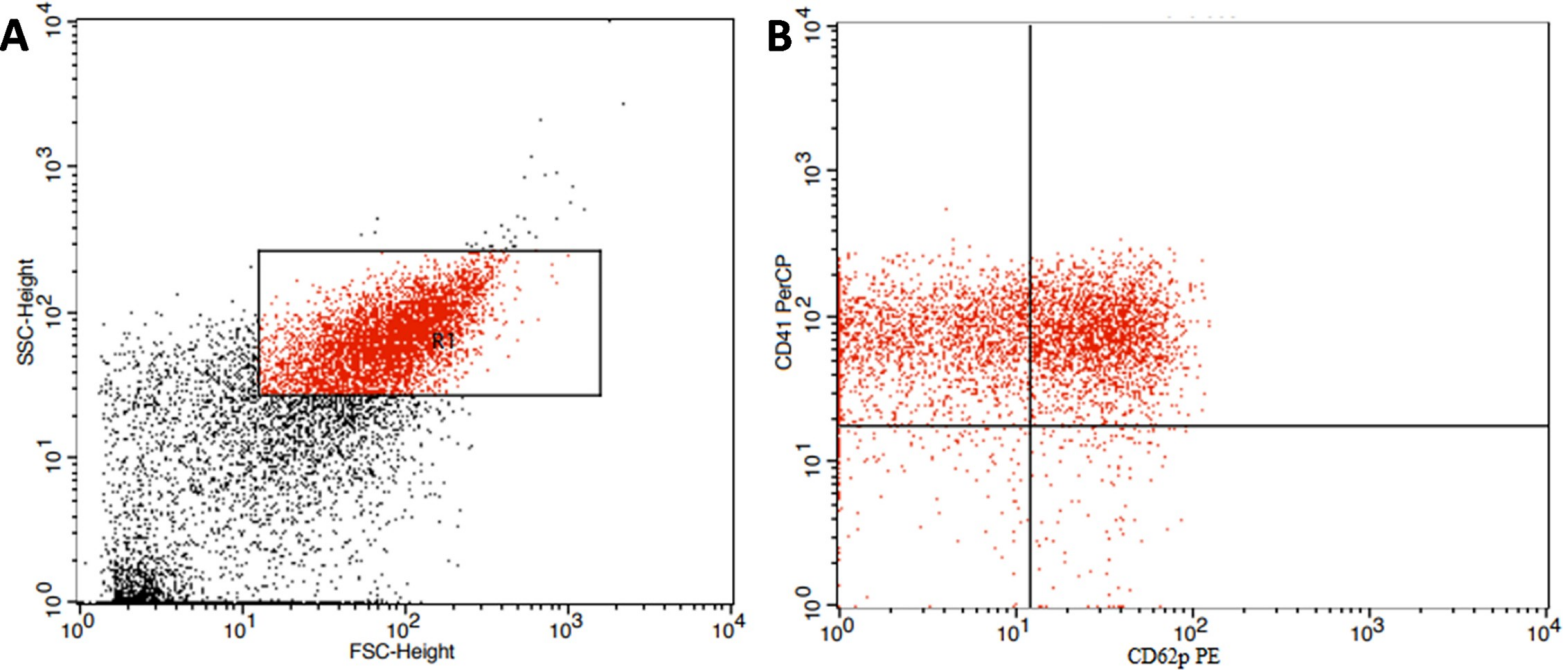
Flow cytometric detection of activated platelets. A:a forward/side scatter plot was used to identify platelets (R1). B: platelets expressing CD62P weremeasuredamong the CD41 positive events (activated platelets).

*Flow cytometric detection of activated platelets*. Fifty μl of blood sample was mixed with 5 μL of (Per-CP) conjugated CD41 PC-309-T100 (EXBIO Praha, a.s, Vestec, Czech Republic), and PE-conjugated CD62p Catalog No.555524 (Becton Dickinson Biosciences, (San Jose, California, USA)). After incubation for 15 min in the dark, and RBCs were then lysed. The cells were washed once then resuspended in PBS, and analyzed by FACSCalibur flow cytometer with CellQuest software. Anti-human IgG was used as an isotype-matched negative control with each sample, "Fig 2".

## Statistics

All data were analyzed using SPSS (version 21) with descriptive stats for calculations of mean, SD, SE, percentages, median, most of data were abnormally distributed as detected normality test of Shapiro-Wilk (p<0.05), (non-parametric data) with skewness of +0.4 (mild asymmetry), and our data were platykurtic (kurtosis = 0.9). Tests for significance included Mann-Whitney U-test was used for two groups of categorical data, while Kruskal Wallis test and one way Anova with post-hoc and LSD were used for more than two groups of categorical data, legacy Chi square test was performed for qualitative data, Spearman correlation was used for association between quantitative data, all data were considered significant at *p*-value <0.05.

## Results

As expected, malignant patients had a mean age higher than non-malignant ones with comparable sex; also they had a significantly higher fever but without difference in fever duration. COVID-19 malignant patients had higher propensity for infection and complication detected in their significant higher score of ground glass oppacities as demonstrated in "Table 1".

**Table 1:** Clinico-radiological presentation of patients infected with covid-19.

Clinical data	COVID-19 malignant (N = 23)	COVID-19 non-malignant (N = 19)	*p*-value
**Age (mean±SE)**	48.6±3.05	30.7±3.03	<0.0001*
**Sex (female/male)**	7/16	3/16	0.3
**contact to cases**	11/23	7/19	0.5
**Temperature**	38.8±0.17	38.1±0.19	0.012*
**Cough**			
**No**	5 (21.7%)	5 (26.3%)	0.7
**positive (dry)**	18 (78.3%)	14 (73.7%)	
**Fever days median**	5.2±0.3	4.3±0.5	0.1
**Dyspnea**			
**grade 0**	4 (17.4%)	4 (21.1%)	
**grade 1**	4 (17.4%)	4 (21.1%)	
**grade 2**	3 (13%)	3 (15.8%)	0.9
**grade 3**	6 (26.1%)	3 (15.8%)	
**grade 4**	6 (26.1%)	5 (21.1%)	
**Diarrhea**			
**no diarrhea**	12 (52.2%)	13 (68.4%)	0.3
**with diarrhea**	11 (47.8%)	6 (31.6%)	
**Anosmia**	10/23	4/19	0.1
**GGO**			
**no involvement (score 0)**	0 (0%)	9 (47.4%)	
**mild (scores 1, 2)**	7 (30.4%)	2 (10.5%)	0.003*
**moderate (scores 3, 4)**	7 (30.4%)	4 (21.1%)	
**severe (score 5)**	9 (39.1%)	4 (21.1%)	

Data expressed as mean ±SE, percentage, Mann Whitney U-test, legacy Chi square test

*;significant

Malignant COVID-19 patients had significantly lower levels of hemoglobin, platelet counts, and albumin levelsthanCOVID-19 non-malignant ones; on the other hand, the formers had significantly higher CRP, LDH, AST, creatinine levels, and higher PI score than COVID-19 non malignant group "Table 2".

**Table 2:** Laboratory data of covid infected patients.

laboratory data	covid malignant	covid non-malignant	*p*-value
**CBC**			
•**HB**	11.4±0.4	14.3±0.3	<0.001*
•**TLC**	6.1±0.7	6.8±0.5	0.5
•**Neutrophil**	4.4±0.7	4.3±0.5	0.5
•**Lymphocyte**	1.2±0.1	1.4±0.1	0.1
•**Eosinophil**	0.24±0.1	0.14±0.03	0.2
•**Platelets**	215.9±19.1	319.3±18.8	<0.001*
**CRP (mg/L)**	43.8±6.4	16.3±4.2	0.001*
**D-dimer (ng/mL)**	790.1±175.6	435.8±64.2	0.08
**Ferritin (ng/mL)**	729.4±116.1	529.6±93.4	0.2
**LDH (U/L)**	407.6±35.2	281.1±117.1	0.009*
**HbA1C**			
**non-diabetic <5.5**	2 (8.7%)	5 (26.3%)	
**controlled 5.5–6.5**	11 (47.8%)	8 (42.1%)	0.3
**uncontrolled >6.5**	10 (43.5%)	6 (31.6%)	
**LFTs**			
**ALT (U/L)**	37.5±7.6	29.2±5.4	0.4
**AST (U/L)**	43.5±8.1	24.6±2.6	0.049*
**Albumin (g/L)**	37.2±1.1	49.1±0.8	<0.001*
**Bilirubin (mg/dL)**	1.03±0.3	075±0.04	0.3
**RFTs**			
**urea (mg/dL)**	33.6±3.03	26.6±2.3	0.08
**creatinine (mg/dL)**	1.1±0.1	0.91±0.03	0.038*
**urea/creatinine ratio**	34.5±6.1	28.9±2.2	0.4
**NLR**	4.02±0.6	3.3±0.4	0.3
**PLR**	203.3±23.5	256.6±29.1	0.2
**PNR**	70.2±12.03	100.0±16.2	0.1
**PI**			
**0**	0 (0%)	0 (0%)	0.016*
**1**	4 (17.4%)	10 (52.6%)	
**2**	19 (82.6%)	9 (47.4%)	

CBC; complete blood count, Hb; hemoglobin, TLC; total leukocytic count, CRP; C-reactive protein, LDH; lactate dehydrogenase,HbA1C; hemoglobin A1C, LFTs; liver function tests, ALT; alanine aminotransferase, AST; aspartate aminotransferase, RFTs; renal function tests, NLR; neutrophil to lymphocyte ratio, PLR; platelet to lymphocyte ratio, PNR; platelet to neutrophil ratio, PI; prognostic index. Data expressed as mean ±SE, number, and percentages, data analyzed using Mann Whitney U-test, Kruskal-Wallis test, Chi square test

*; significant, *P*<0.05.

### Differential expression of microparticles in COVID-19 malignant and non-malignant patients compared to healthy controls

Significant accumulations of TMPs, PMPs, EMPs, and activated platelets in COVID-19 patients compared to healthy controls, and after performing post-hoc analysis with LSD, we found significant difference regarding TMPs and EMPs between COVID-19 malignant patients compared to COVID-19 non-malignant patients (*p* = 0.007, and *p* = 0.002 respectively), and healthy control (*p*<0.0001, and *p*<0.0001 respectively). on the other hand, no significant difference between COVID-19 malignant versus COVID-19 non-malignant patients regarding PMPs (*p* = 0.6), and activated platelets (*p* = 0.3).Meanwhile, significant accumulations of the PMPs and activated platelets in COVID-19 malignant patients compared to healthy controls (*p* = 0.001 and*p*<0.0001 respectively) "Table 3".

**Table 3:** Differential expression of MPs among COVID infected patients and healthy controls.

MPs	COVID-19 malignant (N = 23)	COVID-19 non malignant (N = 19)	Healthy controls (N = 20)	*p* value
**TMPs**	58.76±2.7	67.9 ±2.5	26.0±1.2	<0.0001*
**PMPs**	63.66 ±66	62.01±2.6	53.38 ±2.2	= 0.003*
**EMPs**	15.4±0.84	11.94±0.83	11.26±0.43	<0.0001*
**activated platelets**	29.42±42	24.98±3.9	9.46±1.2	<0.0001*

TMPs total microparticles, PMPs platelet microparticles, EMPs endothelial microparticles.Data expressed as mean ±SE, one way Anova for analysis with post-hoc and LSD, MPs; microparticles

*;significant.

### Correlations between microparticles and laboratory data

We detected significant negative correlations between TMPs and PMPs (r = -0.3, *p* = 0.03), and TMPs with EMPs (r = -0.37, *p* = 0.016), while significant positive correlation between TMPs with D-dimer (r = +0.349, *p* = 0.023), furthermore, significant positive correlation between activated platelets and CRP (r = +0.321, *p* = 0.038), and D-dimer (r = +0.335, *p* = 0.03) were demonstrated in our results "Table 4".

**Table 4:** Correlations between microparticles and laboratory data.

MPs		CRP	D-dimer	ferritin	LDH
**TMPs**	r	.051	.349	-.167	-.213
	p	.749	.023*	.290	.176
**PMPs**	r	.112	-.090	-.004	.233
	p	.481	.571	.982	.138
**Activated plt**	r	.321	.335	.160	.006
	p	.038*	.030*	.312	.970
**EMPs**	r	.016	.058	-.025	.022
	p	.922	.717	.878	.892

TMPs; total microparticles, PMPs; platelet microparticles, EMPs; endothelial microparticles, r; Spearman correlation coefficient, p; significant

*; significant, NA; not applicable.

Distribution of different microparticles and platelet activation according to severity scores of GGO was shown in "Table 5", progressive increase in TMPs, PMPs, EMPs, and platelet activation according to severity of GGO in COVID-19 malignant patiets, while in non-malignant patients, patients with severe GGO had higher mean values of microparticles than patients with lower scores.

**Table 5:** Distribution of MPs according to severities of GGO in COVID-19 malignant and non-malignant patients.

MP	COVID-19 malignant (GGO scores)				COVID non-malignant (GGO scores)			
	score 0	mild	moderate	severe	score 0	mild	moderate	severe
**TMP**	0	52.7±5.2	61.8±4.4	61.2±4.6	71.4±1.3	71.4±2.2	71.8±1.4	54.5±9.4
**PMP**	0	60.7±4.6	64.1±2.5	65.6±2.2	64.5±3.4	71.7±7.7	55.1±7.3	56.6±4.7
**EMP**	0	13.9±0.8	13.7±1.0	17.9±1.7	1.6±0.6	12.2±1.2	11.2±1.1	14.8±3.6
**A.Plt**	0	26.0±6.7	30.6±6.8	31.1±6.5	17.6±5.9	27.1±14.9	18.5±4.7	30.5±11.9
**Total**	0	7	7	9	9	2	4	4

TMPs; total microparticles, PMPs; platelet microparticles, EMPs; endothelial microparticles, GGO; ground glass opacities.

**Fig 3:**
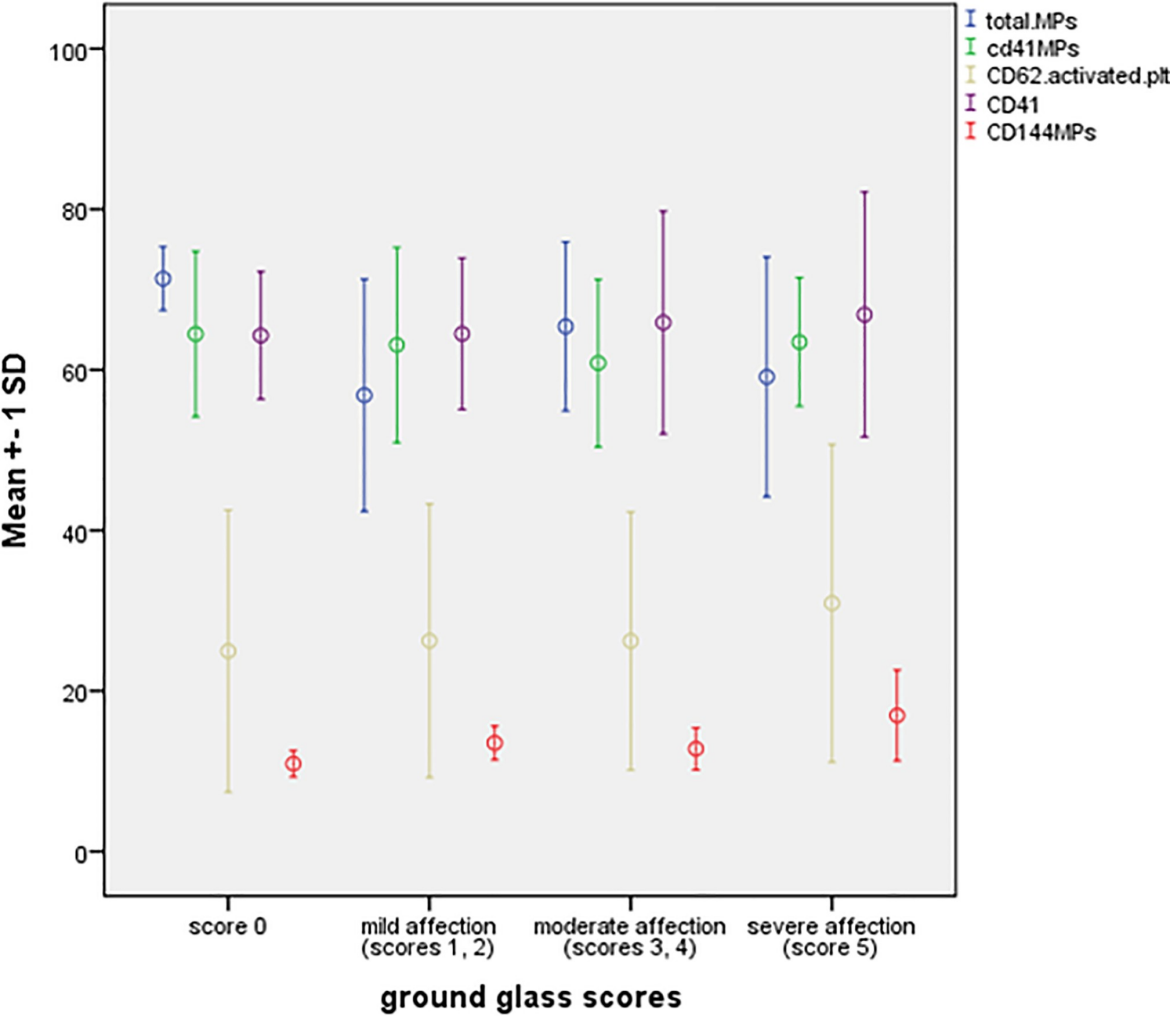
Differences in the mean values of MPs according to GGO score.

Significant accumulation of EMPs in patients with severe GGO compared to those with lower scores (*p* = 0.004), while no significant difference in the mean TMPs (*p* = 0.053), PMPs (*p* = 0.87), and activated platelets (*p* = 0.85), with post hoc analysis and LSD, patients with severe GGO had a significantly higher EMP compared to those with free CT (*p* = 0.001), minimal GGO (*p* = 0.037), and moderate GGO (*p* = 0.009), "Fig 3".

**Fig 4:**
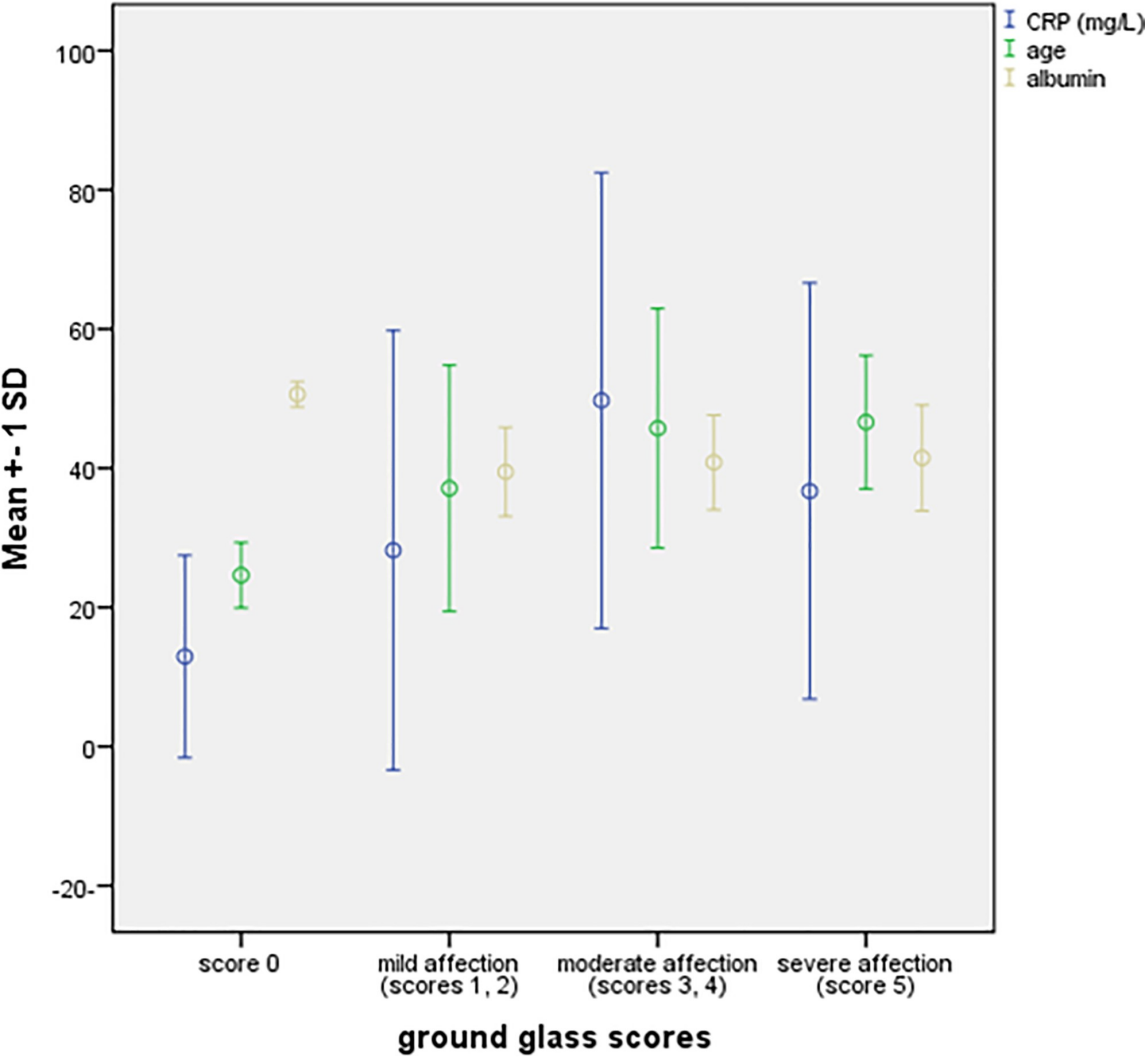
Relations between severities of GGO to hematologic parameters and age.

**Fig 5:**
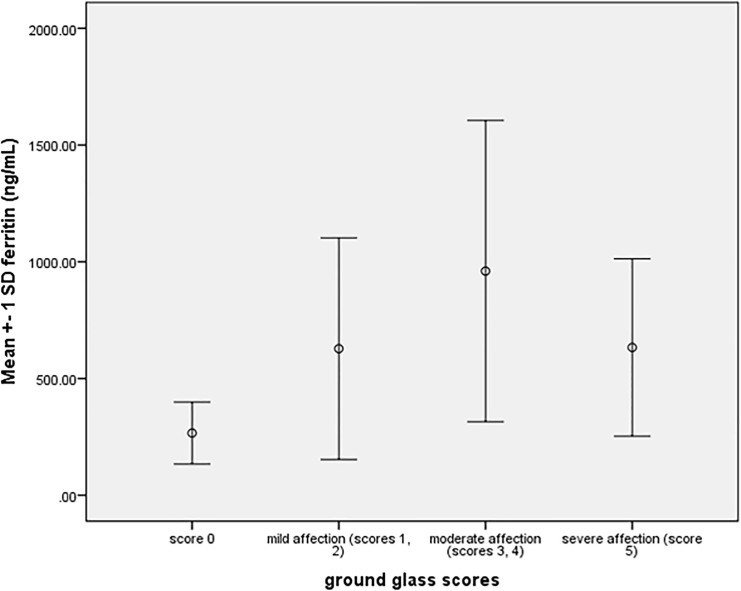
Relation between severities of GGO to ferritin level.

Going through different hematologic parameters and clinical manifestations, we found that age was significantly related to ground glass opacities with severe GGO more evident in older patients (*p* = 0.002), significant higher levels of CRP (*p* = 0.031), and ferritin (*p* = 0.017) were detected in patients with moderate GGO, while albumin was significantly higher in patients with free CTs than those with GGO (*p* = 0.003), post hoc analysis failed to detect association between other hematologic parameters and CT changes except patients with moderate GGO had a significantly higher D-dimer level compared with score 0 (*p* = 0.039), but insignificantly differed compared with other CT changes "Figs 4 and 5".

## Discussion

Corona virus disease 2019 (Covid-19) is a devastating health pandemic pervades the world and causes high mortality rates especially among elderly patients, based on the cells infected by covid-19, the disease passes into 3 clinical stages [21], from an initial asymptomatic state persisting for about 2 days, where the virus binds to ACE2 receptors of ciliated cells of the nasal cavity, then the virus propagates in the subsequent state to upper and conducting airways where it becomes manifested clinically with elevated levels of serum CXCL10 [22], about 80% of covid infected patients have a disease restricted to these states, however unfortunately, twenty percent of patients progress to final stage where the virus infects alveolar type II cells of peripheral and subpleural areas with pulmonary infiltrates rich in hyaline membrane and poor in multinucleated giant cells [23] resulting in diffuse alveolar damage followed by scarring and fibrosis.

Large numbers of studies were conducted to detect the abnormalities of hematologic parameters in covid infected patients, such as low platelets count, low lymphocyte count and percentage, low total protein level, high D-dimer level, high leukocyte count, high C-reactive protein, high creatinine level, high neutrophil count and percentage, high creatine kinase activity, and prolonged prothrombin time [24–27]. Among the prementioned hematologic abnormalities, high D-dimer level, prolonged prothrombin time and low platelet count reflect hypercoagulable state in COVID-19 patients that may finally progress to overt disseminated intravascular coagulation [28].

Our results found that anemia, lymphopenia, esinophilia, elevated CRP, LDH, AST, serum albumin, and creatinine levels, and low PI were significantly higher in COVID-19 malignant patients compared to COVID-19 non malignant patients, furthermore, low platelet count and high D-dimer were encountered in malignant group more than non-malignant group.

One of the hallmarks of viral infection is the formation of inflammatory milieu around infected cells; shedding of MPs can be induced by viral pathogens and are closely related to cell activation or initiation of apoptosis and necrosis in these infected cells [29], Our results showed that patients with COVID-19 had significantly higher levels of MPs whether total, platelet, and endothelial types than healthy donors indicating that they have been induced by COVID-19 infection with significant accumulations of EMP and TMP were detected in covid malignant patients compared to covid non-malignant patients indicating that malignancy is associated with high levels of microparticles.

Undoubtedly, immune-mediated inflammation plays an important role in the pathogenesis of COVID-19 with progressive reduction in lymphocytic count and elevation of neutrophilic count, immune disturbance in covid-19 starts early in infection with progressive reduction in B cells, CD8+, CD4+ T cells, NK cells which precedes radiologic evidence of the disease [23, 30].

Release of interleukin-6 with pulmonary and peripheral endothelial injury, due to viral attack, promotes hypercoagulibilty state in covid infection, in addition to the release of antiphospholipid antibodies with severe immune response to COVID infection results in disturbed coagulation [31], circulating microparticles support the formation of dense fibrin networks formed of thin resistant fibers to enzymatic lysis indirectly through promoting thrombin generation and directly via interaction of MPs with fibrinogen, with better understanding of the mechanisms underlying the formation of lysis-resistant hemostatic fibrin clots and thrombi formed in pathological conditions associated with increased coagulopathy such as covid-19 [32]. The high levels of MPs and activated platelets in our patients, together with the positive relations of TMP and activated platelets with D-dimer may shed the light on potential mechanisms underlying the formation of lysis-resistant hemostatic fibrin clots and thrombin, and pathological conditions associated with increased coagulopathy such as COVID-19 [32].

Multislice CT chest has a sensitivity of 97% and 75% in PCR positive and negative cases respectively with 25% specificity for detection of COVID-19 infection, many studies have reported CT pulmonary changes with COVID-19 [27, 33] which were combined in a prognostic score predicting the mortality in COVID-19 infected patients [34]. The most common observed CT changes were multiple bilateral peripheral GGO [35]. Many reports demonstrated a positive correlation between CT changes and abnormal blood coagulation parameters (based on measurements of platelets and D-dimer) [23, 36, 37], our results agreed with the previous studies where only patients with moderate GGO had a significantly higher D-dimer level compared with free CT, furthermore, our results in alignment with Zhang et al [23], demonstrated an association between CRP, and serum ferritin with the severity of CT changes.

A role of MP in tumor progression has been suggested by both *in vitro* and *in vivo* studies. Evidence exists that MPs of platelet origin are the main players in the process of progression, being rich in pro-angiogenic factors [38], in the current study, we recruited malignant patients with controlled tumors and no signs of tumor progression, we detected no significant differences in MPs of platelet origin between malignant and non-malignant patients, but significantly higher levels of MPs of endothelial origin in malignant patients to suggest that the difference may be due COVID-19 infection.

What is worth mentioning, the association between MPs and CT changes, where we reported an association between EMP and severity of CT changes, and to our knowledge; this was the first study to report this association. Additionally, to minimize the bias of differences in MPs level between study groups, we excluded patients with uncontrolled malignancies as several studies reported significantly higher levels of MPs in these patients than patients with controlled malignancies or control subjects [39].

To date, MPs are critical factors in intercellular communications, and key biomarkers in coagulopathy, inflammation, autoimmune diseases, and cancer, so their detection is important for diagnosis and characterization of an uncommon viral infection such as COVD-19.

### Strengths and limitations of the study

As far as we know, this is the first study addressing the relations of MPs with COVID-19 and may provide some explanations for the hypercoagulable state complicating COVID-19 and increasing mortality among those patients. Still, the study has some drawbacks. A study on a larger population is needed to confirm the results and to better assess the MPs relation with disease severity. Our study included different types of cancers, further studies focusing on certain cancers may give interesting results.

## Conclusion

The study detected significantly higher EMPs and TMPs in malignant patients compared to non-malignant patients with significant accumulation of PMPs, EMPs, TMPs, and activated platelets in COVID-19 patients than healthy controls, circulating MPs and activated platelets may be promising novel prognostic biomarkers capable of identifying potentially severe COVID-19 patients who require immediate care especially in cancer patients.

## Supporting information

S1 File (SAV)

S1 Fig Female patient 60 years axial and coronal CT lung window shows bilateral diffuse ground glass opacities seen involving both lung parenchymas (grade 5) sever type COVID-19. (DOCX)

S2 Fig Female patient 33 years old axial (B&C) and coronal (A) CT lung window show bilateral diffuse ground glass opacities seen involving both lung parenchymas (grade 5) severe type COVID-19. (DOCX)

S3 Fig Female patient 25 years old axial (B&C) and coronal(A) CT lung window shows bilateral diffuse ground glass opacities seen involving both lung parenchymas (grade 4) Moderate type COVID-19. (DOCX)

S4 Fig Female patient 65 years old axial (B&C) and coronal(A) CT lung window shows bilateral diffuse ground glass opacities seen involving both lung parenchymas (grade 2) mild type COVID-19.
(DOCX)
