# Supplementary material for: Circulating microparticles and activated platelets as novel prognostic biomarkers in COVID-19; relation to cancer
Source: PLoS One. 2021 Feb 22;16(2):e0246806. doi: 10.1371/journal.pone.0246806 (PMC7899358; doi:10.1371/journal.pone.0246806)

**S2 A, B, C**

Case (2)

Female patient 33 Yrs old axial (B&C) and coronal (A) CT lung window show bilateral diffuse ground glass opacities seen involving both lung parenchymas (grade 5) severe type COVID-19.

A


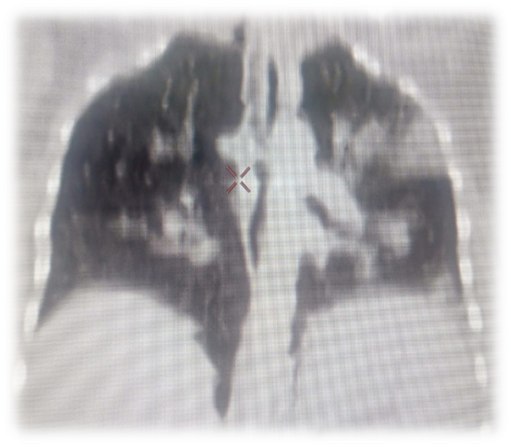


B


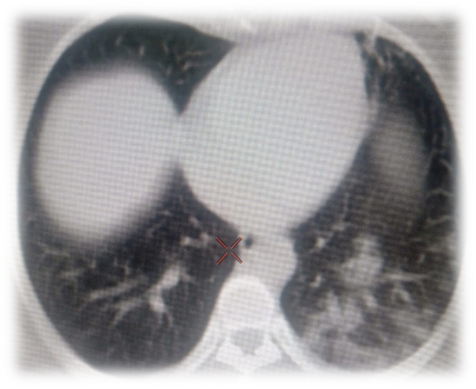


C


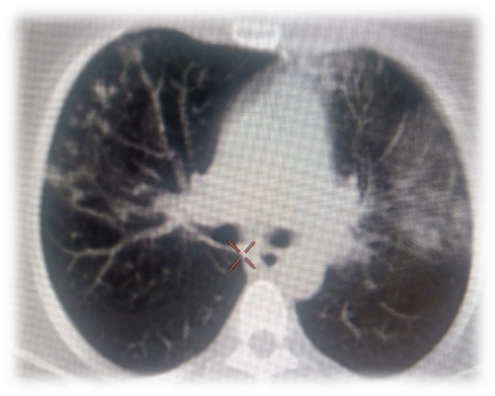

Supplement: S2 Fig — (DOCX) [file pone.0246806.s003.docx]
