# Supplementary material for: Circulating microparticles and activated platelets as novel prognostic biomarkers in COVID-19; relation to cancer
Source: PLoS One. 2021 Feb 22;16(2):e0246806. doi: 10.1371/journal.pone.0246806 (PMC7899358; doi:10.1371/journal.pone.0246806)

**S-3 A, B, C**

Case (3)

Female patient 25 Yrs old axial (B&C) and coronal(A) CT lung window shows bilateral diffuse ground glass opacities seen involving both lung parenchymas (grade 4) Moderate type COVID-19.

A


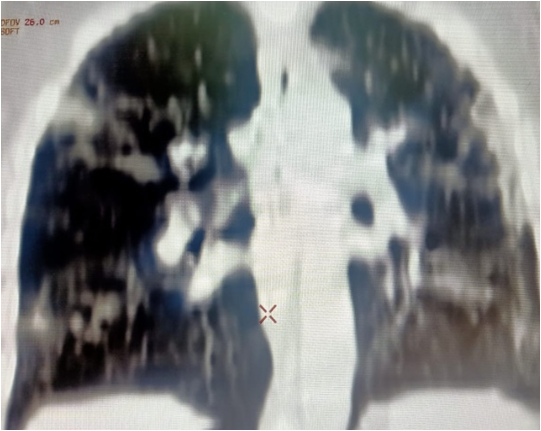


B


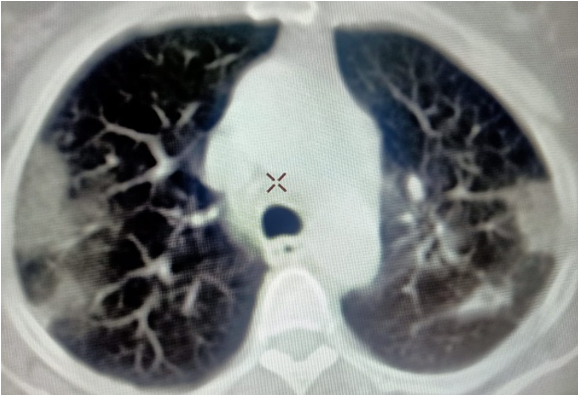


C


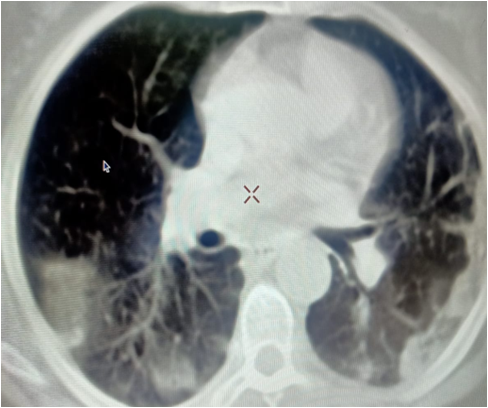

Supplement: S3 Fig — (DOCX) [file pone.0246806.s004.docx]
