# Supplementary material for: Circulating microparticles and activated platelets as novel prognostic biomarkers in COVID-19; relation to cancer
Source: PLoS One. 2021 Feb 22;16(2):e0246806. doi: 10.1371/journal.pone.0246806 (PMC7899358; doi:10.1371/journal.pone.0246806)

**S-4 A, B, C**

Case (4)

Female patient 65 Yrs old axial (B&C) and coronal(A) CT lung window shows bilateral diffuse ground glass opacities seen involving both lung parenchymas (grade 2) mild type COVID-19.

A


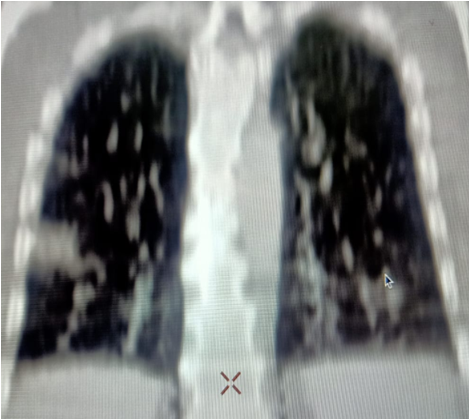


B


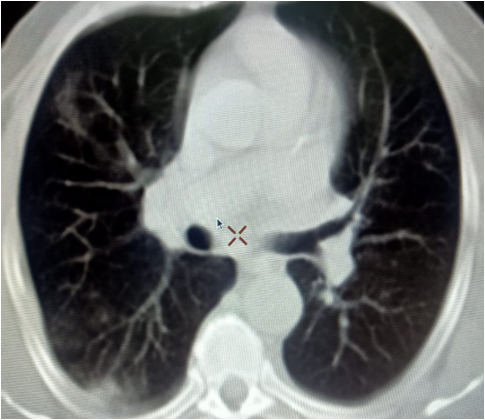


C


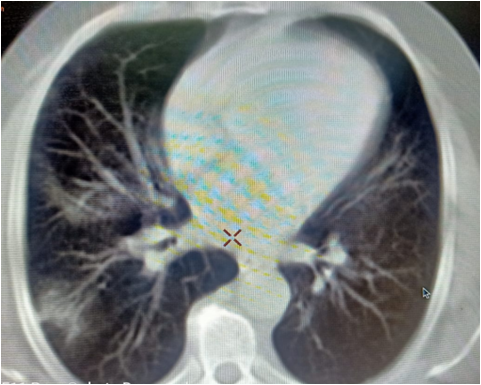

Supplement: S4 Fig — Female patient 65 years old axial (B&C) and coronal(A) CT lung window shows bilateral diffuse ground glass opacities seen involving both lung parenchymas (grade 2) mild type COVID-19. (DOCX) [file pone.0246806.s005.docx]
